# Serological Investigation of Akabane Virus Infection in Cattle and Sheep in Nigeria

**DOI:** 10.1155/2016/2936082

**Published:** 2016-01-26

**Authors:** Daniel Oladimeji Oluwayelu, Comfort Oluladun Aiki-Raji, Emmanuel Chibuzor Umeh, Samat Odunayo Mustapha, Adebowale Idris Adebiyi

**Affiliations:** Department of Veterinary Microbiology and Parasitology, University of Ibadan, Ibadan 20005, Nigeria

## Abstract

Akabane virus (AKAV) is recognized as an important pathogen that causes abortions and congenital malformations in ruminants. However, it has not received adequate attention in Nigeria. Therefore, in investigating this disease, serum samples from 184 (abattoir and farm) head of cattle and 184 intensively reared sheep from two states in southwest Nigeria were screened for antibodies against AKAV using enzyme-linked immunosorbent assay. An overall seropositivity of 70.1% (129/184) was obtained with antibodies being detectable in 73.8% of abattoir (trade) cattle and 40.0% in farm cattle, while 4.3% (8/184) seropositivity was observed in sheep. All the age groups of cattle tested had seropositive animals, 0-1 year (1/7, 14.3%), 2-3 years (17/34, 50.0%), 4-5 years (92/121, 76.0%), and >5 years (19/22, 86.4%), while in sheep only the age groups of 2-3 and 4-5 years showed seropositivity of 4.1% (4/97) and 8.2% (4/49), respectively. The detection of antibody-positive animals among unvaccinated cattle and sheep provides evidence of AKAV infection in Nigeria. These findings call for continuous monitoring of the disease among ruminants in order to ascertain the actual burden and increase awareness of the disease. This will facilitate early detection and aid the development of appropriate control measures against the disease in Nigeria.

## 1. Introduction

The family Bunyaviridae is the largest virus family with over 350 member viruses that are classified into five genera, including the* Orthobunyavirus* genus which contains a large number of viruses that share common genetic features [1]. Based on serological relationships, viruses in this genus have been divided into serogroups with the Simbu serogroup being one of the largest. Several Simbu serogroup viruses isolated from domestic and wild animals as well as mosquito and* Culicoides* vectors have been found in Africa, Australia, Asia, and the Middle East [2–6]. This serogroup, which includes Akabane, Aino, and Shamonda viruses, plays a significant role as pathogens of ruminants with Akabane virus (AKAV) being the most recognized [7]. In particular, Akabane virus, a teratogenic,* Culicoides*-borne virus, replicates in many kinds of natural host species and in several experimental animals [8]. Based on serological evidence, cattle, horses, donkeys, sheep, goats, pigs, camels, and buffaloes appear to be infected in natural situations [9, 10]. AKAV has been identified as a cause of seasonal epizootics of reproductive disorders (abortions, premature births, and stillbirths) and congenital arthrogryposis, hydranencephaly, or microencephaly in cattle, sheep, and goats, sometimes resulting in significant economic losses [1, 6, 11].

Although virus isolation [2], histopathology, immunohistochemistry, serology, and genetic analysis [12] have been used for laboratory confirmation of AKAV infections, serology is the most practical and commonly used technique. Specific antibodies against AKAV have been detected using virus neutralization test (VNT) and enzyme-linked immunosorbent assay (ELISA) [13, 14]. In Africa, limited information based on virus isolation and/or serology revealed the presence of AKAV in Kenya [2, 10], Sudan [15, 16], South Africa [5, 17], Zimbabwe [18], and Tanzania [19] with limited information from Nigeria and perhaps West Africa. In Nigeria, although abortions and congenital malformations associated with AKAV such as arthrogryposis, kyphosis, and scoliosis have been reported in ruminants [20–22], the virus has not received adequate attention as a possible cause of these conditions. Therefore, as part of ongoing surveillance for* Culicoides*-borne arboviruses of livestock in Nigeria, we investigated the presence of specific antibodies against AKAV in cattle and sheep from Oyo and Ogun states, southwest Nigeria.

## 2. Materials and Methods

### 2.1. Study Area

This study was based on a cross-sectional design and conducted between May and September 2015 in Ogun and Oyo states of southwest Nigeria. The study area extended from latitudes 7°00′ to 8°00′ north and longitudes 3°35′ to 4°00′ east. Sera were collected in Oyo state from 184 head of trade cattle at the central municipal abattoirs in Bodija and Ogbomoso and a privately owned farm at Ido where they were kept semi-intensively. Cattle slaughtered at the abattoirs are sourced mainly from the northern part of the country and neighbouring countries, where they were reared on free-range management system that exposes them to insect vectors. Also, 184 sera were collected from semi-intensively reared sheep from farms and backyard flocks in Akinyele, Egbeda, Ido, Ibadan North, and Ona-Ara in Oyo state as well as from Imeko and Odeda in Ogun state. The semi-intensively managed cattle and sheep were allowed to graze on pastures during the day and kept indoors at night. However, the animal houses were not insect-proof.

### 2.2. Sample Collection

Blood (about 5 mL) was collected at slaughter from abattoir cattle while farm cattle and sheep were bled via the jugular vein into sterile plain bottles without anticoagulant. The blood was allowed to clot at room temperature after which sera were decanted into sterile Eppendorf tubes and stored at −20°C until tested.

### 2.3. Detection of AKAV Antibodies by Competitive ELISA

All the collected sera were analyzed using a commercially available ID Screen® competition ELISA kit (IDvet, Montpellier, France) that detects anti-G1 antibodies directed against AKAV in ruminant serum and plasma. The test, which was reported by the manufacturer to lack cross-reactivity with other viruses in the* Bunyaviridae* family, such as Schmallenberg virus (SBV), Rift Valley fever virus (RVFV), and Aino virus, and to have a high (96.52%) correlation with the VNT, was performed according to the kit protocol. For each sample, results were expressed as sample/negative percentage (S/N%) using the optical densities (OD) from the ELISA reader: S/N% = OD_sample_/OD_negative  control_ × 100. Samples that presented S/N% lower than 30%, between 30% and 40%, and >40% were considered positive, doubtful, and negative, respectively.

### 2.4. Statistical Analysis

Results of serology were analyzed using the statistical package GraphPad Prism version 5.01 (San Diego, USA). Differences in AKAV antibody seroprevalence between cattle and sheep, abattoir and farm cattle, and male and female animals were evaluated using chi-square (*χ* ^2^) test. Furthermore, seroprevalence results based on breed, age group, and location were subjected to one-way ANOVA and subsequently to Tukey's posttest for performing multiple comparisons in order to assess statistically significant differences between all possible pairs of groups. The level of statistical significance was *P* < 0.05.

## 3. Results

Out of 184 sheep sera tested, 8 (4.4%), 22 (12.0%), and 154 (83.7%) were positive, doubtful, and negative, respectively, for AKAV anti-G1 antibodies while, of the 184 cattle sera tested, 129 (70.1%), 37 (20.1%), and 18 (9.8%) were positive, doubtful, and negative, respectively (Table 1). The prevalence of AKAV antibodies was significantly (*P* < 0.005) higher in abattoir cattle compared to farm cattle (Table 2). For the abattoir cattle, AKAV antibody prevalence was significantly higher in Bodija central abattoir compared to the Ogbomosho abattoir. Overall, based on cattle breed, AKAV antibody prevalence was 66.4% (75/113), 77.8% (28/36), 75.0% (3/4), 83.3% (10/12), and 76.5% (13/17) for White Fulani, Sokoto Gudali, Kuri, Red Bororo, and crossbreeds, respectively. However, comparison of the AKAV seroprevalence rates in sheep based on age, sex, breed, and location of sample collection revealed no significant differences.

## 4. Discussion

Akabane virus has been shown to be an important pathogen causing abortions and congenital malformations in ruminants [23]. Antibodies to this* Culicoides*-borne, Simbu serogroup virus have been found in ruminants from countries of Australia, Asia, Southeast Asia, the Middle East, and Africa [6, 10, 16, 24, 25]. However, since the initial isolation of Simbu group viruses from cattle and* Culicoides* biting midges in Nigeria [26, 27], there has been no report on the presence of AKAV in Nigeria. In the present study, which is part of ongoing surveillance for* Culicoides*-borne arboviruses in Nigeria [28, 29], AKAV antibody prevalence of 70.1% and 4.3%, respectively, was obtained for cattle and sheep in two states (Ogun and Oyo) of southwest Nigeria using a competition ELISA. According to the internal validation report of the kit manufacturer on the assay, known SBV-, RVFV-, and Aino virus-positive sera produced negative results with the AKAV ELISA kit, demonstrating the absence of cross-reactions with antisera against these viruses. To our knowledge, this is the first report of AKAV antibody-positive animals in Nigeria. As AKAV vaccines are currently not administered to livestock in the country, the detection of seropositive cattle and sheep in this study suggests natural exposure of these animals to AKAV. The higher seroprevalence obtained for cattle, which is similar to the findings of a recent study in Sudan [16], is an indication that they were more likely to be infected with AKAV than sheep. This possibility is buttressed by the knowledge that cattle are allowed to graze more extensively, thus making them more exposed to the* Culicoides* vectors of AKAV than sheep. Moreover, it has previously been reported [30] that some* Culicoides* species have distinct feeding preference for cattle over sheep even when kept in the same vicinity.

Furthermore, it was observed in this study that AKAV antibodies were more prevalent in trade cattle from abattoirs than in farm cattle. This higher seroprevalence may be due to the fact that, compared to farm cattle which are domiciled in one location, trade cattle for slaughter at these abattoirs are sourced from different parts of Nigeria and neighbouring countries where they are kept mostly on free-range. This makes the abattoirs convergence points for animals from diverse geographical locations where they could have had contact with infected* Culicoides* vectors. Since there is unregulated transborder movement of ruminants from neighbouring West African countries into Nigeria, it is possible that some of the seropositive cattle could have imported the disease into the country. Also, the detection of seropositive animals among trade cattle of different breeds suggests that the disease is not breed-restricted.

Previous reports of congenital malformations in cattle and sheep in Nigeria suggested that these defects have no clearly established cause [20–22, 31]. However, the increased prevalence of AKAV antibodies observed with increasing age of cattle and sheep in this study (Table 2) suggests AKAV as a possible cause of these conditions. Since it has been reported that inapparent Akabane infection in adults can lead to abortions, stillbirth, and congenital defects in newborns [10, 32], this finding suggests that AKAV may significantly increase abortion risk among adult cattle and sheep, thus constituting a potential health risk to the animals. Therefore, the findings of this study corroborate the fact that AKAV is endemic in sub-Saharan Africa and underscore the need for large-scale surveillance of the virus in Nigeria in order to establish its role as a possible cause of economic losses in ruminants through abortions, stillbirths, and congenital malformations and develop effective prevention and control strategies. Additionally, virus isolation and genetic characterization of isolates will be essential for understanding the molecular epidemiology and evolution of AKAV in Nigeria.

## Figures and Tables

**Table 1:** Prevalence of AKAV antibodies in sheep and cattle in the study area.

	Number sampled	Positive (%)	Doubtful (%)	Negative (%)
Sheep	184	8 (4.4)	22 (12.0)	154 (83.7)
Cattle	184	129 (70.1)^*∗*^	37 (20.1)	18 (9.8)
Total	364	137 (37.6)	59 (16.2)	172 (47.3)

^*∗*^ *P* = 0.0001.

**Table 2:** Seroprevalence of AKAV in cattle in the study area.

Variable	Group	Number sampled	Positive (%)
Sex	Female	131	92 (70.2)
	Male	53	37 (69.8)

Age (years)	0-1	7	1 (14.3)^*∗*^
	2-3	34	17 (50.0)
	4-5	121	92 (76.0)
	>5	22	19 (86.4)^*∗*^

Source	Abattoir	164	121 (73.8)^*∗*^
	Farm	20	8 (40.0)

^*∗*^ *P* = 0.0001.

## References

[1] MacLachlan N. J., Dubovi E. J. (2011). Bunyaviridae. *Fenner's Veterinary Virology*.

[2] Metselaar D., Robin Y. (1976). Akabane virus isolated in Kenya. *Veterinary Record*.

[3] Hartley W. J., Wanner R. A. (1974). Bovine congenital arthrogryposis in New South Wales. *Australian Veterinary Journal*.

[4] Miura Y., Inaba Y., Tsuda T. (1982). A survey of antibodies to arthropod-borne viruses in Indonesian cattle. *Japanese Journal of Veterinary Science*.

[5] Zeller H., Bouloy M. (2000). Infections by viruses of the families *Bunyaviridae* and *Filoviridae*. *Revue Scientifique et Technique*.

[6] Markusfield O., Mayer E. (1971). An arthrogryposis and hydranencephaly syndrome in calves in Israel, 1969-70: epidemiological and clinical aspects. *Refuah Veterinarith*.

[7] St George T. D., Kirkland P. D., Coetzer J. A. W., Tustin R. C. (2004). Diseases caused by Akabane and related Simbu-group viruses. *Infectious Diseases of Livestock*.

[8] Huang C.-C., Huang T.-S., Deng M.-C., Jong M.-H., Lin S.-Y. (2003). Natural infections of pigs with Akabane virus. *Veterinary Microbiology*.

[9] Al-Busaidy S., Hamblin C., Taylor W. P. (1987). Neutralising antibodies to Akabane virus in free-living wild animals in Africa. *Tropical Animal Health and Production*.

[10] Davies F. G., Jessett D. M. (1985). A study of the host range and distribution of antibody to Akabane virus (genus bunyavirus, family *Bunyaviridae*) in Kenya. *Journal of Hygiene*.

[11] Kurogi H., Inaba Y., Goto Y. (1975). Serologic evidence for etiologic role of Akabane virus in epizootic abortion-arthrogryposis-hydranencephaly in cattle in Japan, 1972–1974. *Archives of Virology*.

[12] Oem J.-K., Yoon H.-J., Kim H.-R. (2012). Genetic and pathogenic characterization of Akabane viruses isolated from cattle with encephalomyelitis in Korea. *Veterinary Microbiology*.

[13] Miura Y., Hayashi S., Ishihara T., Inaba Y., Omori T., Matumoto M. (1974). Neutralizing antibody against Akabane virus in precolostral sera from calves with congenital arthrogryposis-hydranencephaly syndrome. *Archiv für die Gesamte Virusforschung*.

[14] Tsuda T., Yoshida K., Yanase T., Ohashi S., Yamakawa M. (2004). Competitive enzyme-linked immunosorbent assay for the detection of the antibodies specific to Akabane virus. *Journal of Veterinary Diagnostic Investigation*.

[15] Mohamed M. E., Mellor P. S., Taylor W. P. (1996). Akabane virus: serological survey of antibodies in livestock in the Sudan. *Revue d'Elevage et de Médecine Vétérinaire des pays Tropicaux*.

[16] Elhassan A. M., Mansour M. E. A., Shamon A. A. A., El Hussein A. M. (2014). A serological survey of Akabane virus infection in cattle in Sudan. *ISRN Veterinary Science*.

[17] Theodoridis A., Nevill E. M., Els H. J., Boshoff S. T. (1979). Viruses isolated from Culicoides midges in South Africa during unsuccessful attempts to isolate bovine ephemeral fever virus. *Onderstepoort Journal of Veterinary Research*.

[18] Blackburn N. K., Searle I., Phelps B. J. (1985). Viruses isolated from *Culicoides* (Diptera: Ceratopogonidae) caught at the veterinary research farm, Mazowe, Zimbabwe. *Journal of the Entomological Society of South Africa*.

[19] Mathew C., Klevar S., Elbers A. R. W. (2015). Detection of serum neutralizing antibodies to Simbu sero-group viruses in cattle in Tanzania. *BMC Veterinary Research*.

[20] Ate I. U., Allam L. (2002). Multiple congenital skeletal malformations in a lamb associated with dystocia in a Yankasa ewe. *Nigerian Veterinary Journal*.

[21] Bukar M. M., Waziri M., Ibrahim U. I. (2006). Dystocia due to arthrogryposis and associated with a mummified twin in a crossed (Yankassa/Uda) ewe: a case report. *Tropical Veterinarian*.

[22] Ibrahim N. D., Adamu S., Useh S. M. (2006). Multiple congenital defects in a Bunaji bull. *Nigerian Veterinary Journal*.

[23] Coverdale O. R., Cybinski D. H., St George T. D. (1978). Congenital abnormalities in calves associated with Akabane virus and Aino virus. *Australian Veterinary Journal*.

[24] Hartley W. J., Wanner R. A., Della-Porta A. J., Snowdon W. A. (1975). Serological evidence for the association of Akabane virus with epizootic bovine congenital arthrogryposis and hydranencephaly syndromes in New South Wales. *Australian Veterinary Journal*.

[25] Al-Busaidy S. M., Mellor P. S., Taylor W. P. (1988). Prevalence of neutralising antibodies to Akabane virus in the Arabian Peninsula. *Veterinary Microbiology*.

[26] Causey O. R., Kemp G. E., Causey C. E., Lee V. H. (1972). Isolation of Simbu-group viruses in Ibadan, Nigeria 1964–69, including the new types Sango, Shamonda, Sabo and Shuni. *Annals of Tropical Medicine and Parasitology*.

[27] Lee V. H. (1979). Isolation of viruses from field populations of *Culicoides* (Diptera: Ceratopogonidae) in Nigeria. *Journal of Medical Entomology*.

[28] Oluwayelu D. O., Olatoye O., Akanbi M., Hoffmann B. (2011). Seroprevalence of bluetongue virus infection in Oyo state, Nigeria. *Journal of Commonwealth Veterinary Association*.

[29] Oluwayelu D. O., Meseko C. O., Adebiyi A. I. (2015). Serological screening for Schmallenberg virus in exotic and indigenous cattle in Nigeria. *Sokoto Journal of Veterinary Sciences*.

[30] Bartsch S., Bauer B., Wiemann A., Clausen P.-H., Steuber S. (2009). Feeding patterns of biting midges of the *Culicoides obsoletus* and *Culicoides pulicaris* groups on selected farms in Brandenburg, Germany. *Parasitology Research*.

[31] Bello A. A., Nwanena I. A., Hamman I., Aba C. T. (2006). Foetal monster in a four-year old Yankassa ewe with dystocia: a case report. *Tropical Veterinarian*.

[32] Kirkland P. D., Barry R. D., Harper P. A., Zelski R. Z. (1988). The development of Akabane virus-induced congenital abnormalities in cattle. *Veterinary Record*.

